# The perceptions and experiences of medical students in a pediatric buddy program: a qualitative study

**DOI:** 10.1186/s12909-022-03306-w

**Published:** 2022-04-04

**Authors:** Candace Nayman, Jeffrey Do, Alexa Goodbaum, Kaylee Eady, Katherine Moreau

**Affiliations:** 1 Faculty of Medicine, University of Ottawa, Roger Guindon Hall, 451 Smyth Road #2044, Ottawa, ON K1H 8M5 Canada; 2 Centre for Research On Educational and Community Services, University of Ottawa, 136 Jean-Jacques-Lussier Private, Ottawa, ON K1N 6N5 Canada; 3 Faculty of Education, University of Ottawa, 136 Jean-Jacques-Lussier Private, Ottawa, ON K1N 6N5 Canada

**Keywords:** Partnership program, Longitudinal, Medical education, Pediatrics

## Abstract

**Background:**

Partnership programs between medical students and patients provide students with non-clinical experiences that enhance medical learning, especially with respect to humanistic care. We explored the perceptions and experiences of medical students in a pediatric oncology buddy program.

**Methods:**

Using a basic interpretive qualitative approach, we conducted interviews with 15 medical students at three time points: before meeting his/her buddy (pre-interview), four months into the partnership (4-month interview), and at the end of the partnership (post interview). We then conducted a thematic analysis of the interview data.

**Results:**

All students in the program who met the study criteria (*N* = 15/16) participated. The medical students highlighted that: (a) providing support to buddies and their families is important; (b) providing care to children with serious illnesses is emotionally difficult; (c) developing deep connections with buddies and their families is rewarding; and (d) gaining empathy and personal fulfillment from buddies and their families is inevitable.

**Conclusions:**

This study provides an understanding of medical students’ perceptions and experiences in a pediatric oncology, non-clinical buddy program. Tailored one-on-one partnerships between medical students and pediatric oncology patients play an important role in medical education and contributes to the teaching of humanistic care.

## Background

While anatomy, physiology, and pharmacology are tenets of medical education, physicians must be able to connect with patients to provide humanistic care. Humanistic care emphasizes the treatment of each patient as a person, rather than a disease. It is grounded in compassion, caring, sincerity, and integrity [1]. Research demonstrates that early training in humanistic care improves trainees’ clinical performance, communication between physicians and patients, and may even influence the amount of hospital resources they use [1–4].

Non-clinical partnership programs that pair healthcare students with patients can enhance students’ understanding and provision of humanistic care [5–8]. These programs, which can be found in various settings, also help address patients’ psychosocial and spiritual needs [9]. For example, studies where medical students conducted home visits with patients have shown that students who participated in these visits were more likely to identify ethical dilemmas and consider patients’ spiritual needs than those who did not participate [5, 6]. Partnership programs also provide students with opportunities to understand the emotional aspects of living with illnesses, as students spend quality time with patients and often develop meaningful friendships [8]. Through these experiences, students can increase their knowledge, confidence, health literacy, communication and learn first-hand about treating the whole patient [10]. Researchers have called for the integration of formal knowledge and experiential learning and suggested including longitudinal connections with patients in medical training, particularly for improving physician preparation and achieving academic excellence [11].

Despite these documented benefits and calls for reform, research is limited in its exploration of medical students’ perceptions and experiences in non-clinical partnership programs. One study eloquently showed how real-patient learning may give students enhanced confidence, a sense of professional identity, and an appreciation for patient complexity [12]. However, there was no mention of the age of the patients or what criteria were used to pair students with the patients. An exploration of students’ perceptions and experiences in these programs, with detailed information about the pairing process can provide valuable insights into the relationships garnered between students and patients, and, often in pediatrics, their families. It can also uncover students’ motivations for participating in the programs and the lessons they learned through their participation, especially regarding humanistic care, which can then be applied to future clinical practice. Thus, our study sought to answer the following research question: What are the perceptions and experiences of medical students who participate in the pediatric buddy program at the University of Ottawa, Canada?

## Methods

### Setting

The study was conducted at the University of Ottawa from September 2018 to June 2019. It focused on a partnership program offered jointly by the medical school and a pediatric academic hospital. The program, which has operated for more than 20 years, aims to foster nurturing relationships between pediatric oncology patients and first and second year (“pre-clerkship”) medical students. Pre-clerkship students are matched with pediatric oncology patients as “buddies” based on personality traits, interests, and primary language. Students can participate electively in the program during their first two years of medical school, recognizing that their academic workload increases at the beginning of third year, which may impact their participation.

Each year, the program coordinators invite all pre-clerkship students (approximately 328 students) to apply for the program by giving an information session detailing the program and its objectives. Two of the pediatric nurses involved with the program also invite eligible oncology patients (infant to 18 years old) and their parents to join the program. The program coordinators then invite selected students to participate in an interview to determine if there is a patient in the program that would be a suitable match. If a student’s personality and interests matched those of a participating patient, based on intake interviews that assess hobbies and interests, s/he joins the program and is paired with the buddy. Enrollment in the program is performed on an ongoing basis throughout each academic year. In this partnership program, the students and their buddies can do a wide range of activities, including arts and crafts, movie-watching, sporting events, or playground activities. However, chosen activities depend on the interests and capabilities of the patients, as well as their individual hospital protocols. Students meet with their buddies approximately once per week throughout the academic year, although there is no formal time requirement.

### Approach

Informed by the work of Merriam and Grenier [13], we used a basic interpretive qualitative approach, within a constructivist paradigm. We viewed the program from the students’ perspectives and aimed to inductively share the way they interpreted their perceptions and experiences of it. Notably, we were interested in how the students perceived, experienced, and interacted with the pediatric patients in the context of the buddy program. We also acknowledged that data interpretations were mediated through our personal perceptions and experiences with the phenomenon. Specifically, the authors (CN, JD & AG) who conducted the one-on-one interviews with the medical students were previous buddies in the program and, at the time of data collection, medical students. Moreover, the remaining two authors (KE & KM), who assisted with data analysis and interpretation, are medical education researchers with patient engagement interests.

We conducted interviews with each student at the following time points: (1) before meeting his/her buddy (pre-interview); (2) four months into the partnership (4-month interview); and (3) at the end of the partnership (post interview). Our aim was to present the overall meaning of the perceptions and experiences across all students. This approach allowed us to search for commonalities in their perceptions and experiences in the program. We obtained ethics approval from the University of Ottawa Research Ethics Board.

### Participants

Only medical students selected to be in the partnership program in the 2018–2019 academic year who had not yet begun the partnership with their buddies were eligible. One student who had been in a partnership previously was excluded, as this student already had program experience.

### Data collection

The program coordinators circulated an information letter by email to eligible participants. Interested medical students from the program then replied to schedule an interview, where one of the coordinators reviewed the informed consent forms with them and obtained their written consent. Authors (CN, JD & AG) conducted one-on-one interviews with the medical students. While we randomly selected the interviewer for each student, the interviewer remained the same for the student’s pre-, 4-month, and post interviews. The interview guides consisted of semi-structured questions to encourage students to openly discuss their perceptions and experiences (see Table 1). Interviews took place in-person in a private room. All interviews were audio-recorded and transcribed verbatim. The interviews ranged from 18–47 min in length, with pre-match interviews being shorter as participants had not yet experienced the program. Given the potential of the students experiencing psychological discomfort while reflecting on their perceptions and experiences in the program, we offered them access to psychological services through the University’s Student Affairs Office.

**Table 1:** Question prompts used at each interview time point

Pre-Interview	4-month-Interview	Post Interview
1. Describe what you think your role will be	1. Describe what your role has been	1. Describe what your role was
2. What do you hope to experience?	2. How do you think this program has been impacting your buddy and his/her family?	2. How do you think this program impacted your buddy and his/her family?
3. Why do you think your experiences will be this way?	3. What have you been experiencing and learning?	3. What did you experience and learn?
4. What challenges do you anticipate encountering?	4. Why do you think your experiences have been this way?	4. Why do you think your experiences were this way?
	5. What are some challenges you have been encountering?	5. What are some challenges you encountered in the program?

### Data analysis

The goal of the analysis was to identify themes present in more than one interview. First, the authors who conducted the interviews independently read the transcripts. They each took notes on the transcripts and used their notes to inductively create coding schemes for the data. They then used their schemes to independently code the data. Next, they met to review and revise their coding. Specifically, they compared their schemes and coding and, when necessary, re-read and re-coded selected transcripts. Throughout the process, they resolved disagreements through discussion. They also met with the other two authors, who are versed in qualitative methodology and the topic, to discuss their analyses as well as the interpretation of the data. As a team, we then developed descriptions of each theme and identified exemplar quotations for reporting purposes. Throughout the process, we noted our thought processes and decision-making. These notes combined with our use of multiple coders and debriefing discussions enhanced the trustworthiness of the analysis.

## Results

**Fig. 1:**
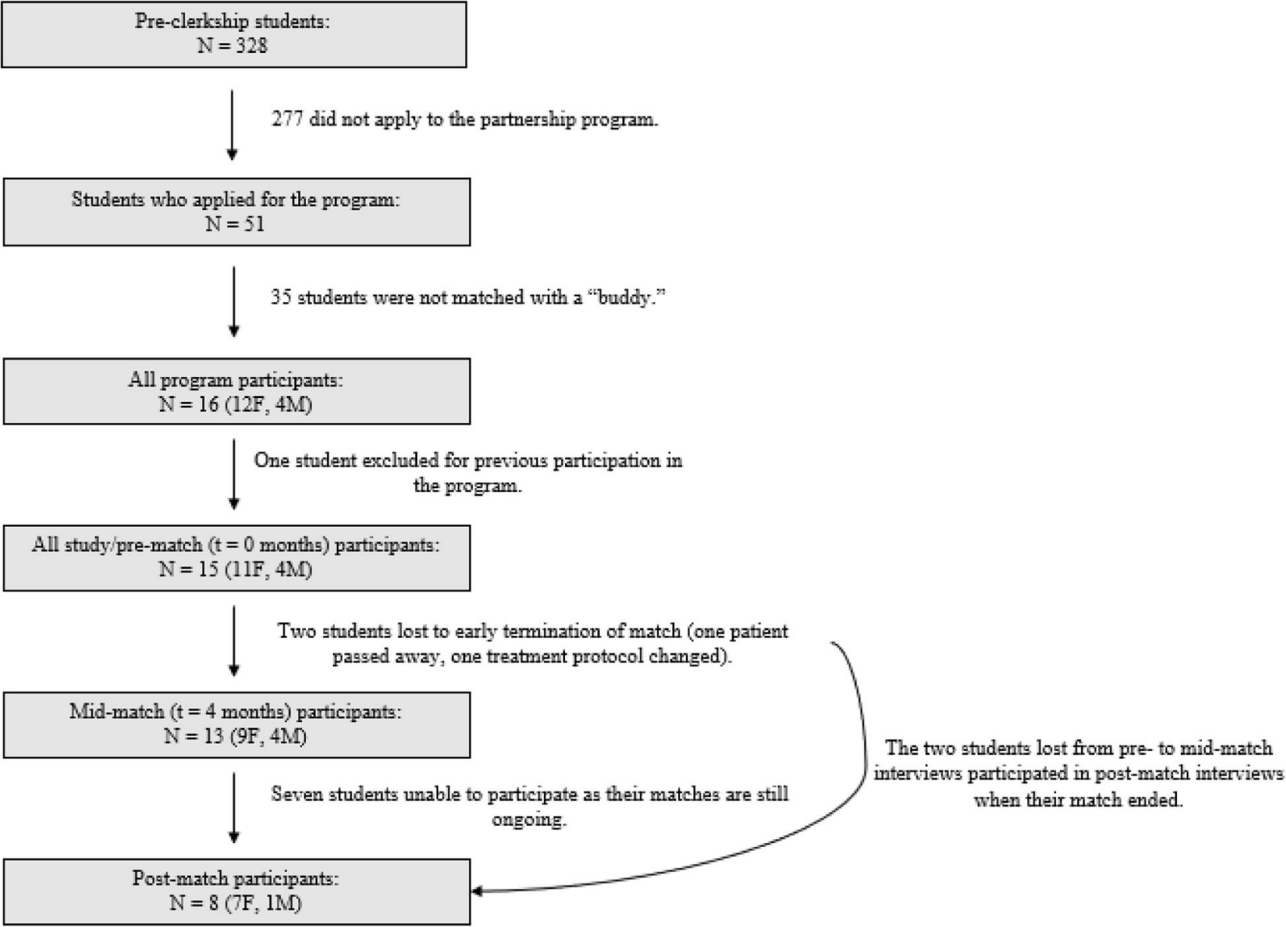
Study timeline and interview participation at each interview time point

Of the 328 pre-clerkship students (F = 164, M = 164) in 2018, 51 applied to the program. Of these students, 16 were selected to be matched with a buddy based on mutual interests and buddy availability. Fifteen (F = 11, M = 4) of the 16 matched medical students in the program met the study inclusion criteria. All eligible students agreed to participate. The students, who all identified as female, had prior experience working with children including, but not limited to, working at summer camps, sports camps, or with formal mentorship organizations. None had experience with pediatric oncology patients. All students participated in the pre-interview, 13 (F = 9, M = 4) students participated in the 4-month interview, and eight (F = 7, M = 1) students participated in the post interview. The remaining seven students still had ongoing matches at the time of the study’s conclusion, as they were first year students and their buddies were still receiving treatment. Figure 1 depicts student participation at each time point.

We identified four themes based on the interview data.

### Providing support to buddies and their families is important

#### Pre-match interviews

Students thought they would help their buddies and families by providing emotional and social support as well as respite. The students predicted that families would struggle with their children’s diagnoses and expressed that they wanted to help. For instance, reflecting on the help she would provide to families, a student stated, “I think my role as buddy will be to support the family primarily” (MS02). For students, helping the families also meant that they would provide practical support by allowing parents to leave the hospital to run errands or to take care of themselves and their other children. The students thought that they would provide emotional support by being available to talk with the buddies and their families, and by being a friend to their buddy. As one student summarized, “just being a person to hang out with them, spend some time with them, as a friend, and take their mind off of what is happening to them right now” (MS06). They anticipated playing games with their buddies and providing new entertainment for them. Students’ expectations of their roles also went beyond simply that of an “entertainer” (MS03). The students thought they would focus on ensuring their buddies and families were comfortable with them as well as with the activities they were doing. Many students stated that the hypothesis of what role(s) they would play developed during the program’s information sessions, both during the initial recruitment of medical students and in an orientation to the program.

#### 4-month and post interviews

Students confirmed that they had in fact experienced the above-mentioned roles, but they noted that they supported their buddies and families more than anticipated in terms of the amount of time they spent with their buddies and families. They confirmed that they were not only their buddies’ companions, but that they also provided much-needed respite for families. A student summarized this idea by stating:

“I think probably one of the most important things that my time with my buddy has been able to show my family is that it’s okay to take a break and that even though your kid’s sick, you don’t have to be the one that’s there for them every single minute of the day.” (MS02 4-month interview)

Students texted their buddies and families to regularly check-in. Students discussed that families were grateful for the partnerships as it allowed them to tend to other responsibilities and to take mental breaks by knowing someone was with their child. Many students were surprised by the impact of their support on parents and how much it helped the families. The students played games and instruments, watched movies, baked, and/or did arts and crafts with their buddies. When reflecting on the experience from the parents’ perspectives, students echoed thoughts such as the following:

“I think they’re just happy to have someone else on their team and someone who can kind of, get her to come out of the house when she’s feeling good, and do things that maybe she doesn’t want to do with her- her mom or dad, but someone who’s more like a friend.” (MS07, post interview)

Students were grateful to have provided new activities to help boost their buddies’ morale, while also being a friend throughout their medical journeys. As a student shared, the patient’s mom “told me, she said that this was definitely like one of the good things to come out of her diagnosis and everything that she’s been going through over the past year (MS07, post interview). The students also noted that they had become close friends with their buddies and that the buddies looked forward to their encounters. They noted how they had become “a support person” and “a fun outlet” (MS06 4-month interview). Students noted that, "it’s been awesome, honestly… just like thinking now like how big of an impact [the patient] had on my life, in just a couple months” (MS07, 4-month interview).

### Providing care to children with serious illnesses is emotionally difficult

#### Pre-match interviews

Before meeting their buddies, students primarily worried about the emotional toll of working with oncology patients. They predicted that they would struggle with families receiving bad news as well as with trying to remain positive for their buddies. For example, a student said:

“I think a challenge will definitely be just the nature of the role, like, these children are sick and they’re going through difficult times, so dealing with, um, like, negative emotions as they come up, and trying to help your buddy stay positive while at the same time acknowledging their, um, like, their stressors, what’s scaring them, and trying to maintain that balance.” (MS06).

Whereas another noted, “the biggest one will probably be just seeing, you know, a kid who’s sick. Like, seeing them go through, you know, if they’re having a bad day, or if we have to cancel something cause they’re not feeling well” (MS07). Students voiced that this anticipated struggle stemmed from a lack of personal experience and exposure to children with cancer, and that this might affect their abilities to relate to families and understand their unique needs, including what they may be experiencing. They also feared the emotional toll of losing a child if their buddies passed away, particularly once they formed close relationships. Some students explained that they had lost family members themselves and that they were worried that the loss of their buddies could resurface their own feelings.

#### 4-month and post interviews

Students confirmed the predicted emotional toll of working with children with serious illnesses:

“Probably the biggest issue was ... remembering the fact that…. I’m her friend because she’s involved in this program, and she’s in this program because she does have a cancer diagnosis ...Even when we’re out having fun, she could text me that night and say that she got some bad news or that she wasn’t feeling well…. That’s kind of at the centre of all of this is that she’s- she’s going through that…. And dealing with the emotions that...come with something like that.” (MS07, post interview)

Some students were tearful during interviews and discussed emotional hardships, including feeling withdrawn in their personal lives. For example, a student realized, “that when I would come home from the buddy experiences, I’d kind of like, I dunno, feel kind of, off? I didn’t feel like doing anything and I couldn’t stop thinking that it’s just so not fair that she’s going through this” (MS01, 4-month interview). Students explained that even on happy days, they knew their buddies were in the program because of their cancer diagnoses and that bad news could arise unexpectedly. They also repeatedly mentioned that, “it’s also challenging to see a little kid have to go through that and to kind of watch the parents explain [that] to the [other] kid that the other little girl isn’t going to get better” (MS02, 4-month interview). Moreover, some students experienced their buddies in critical condition or passing away and noted the emotional difficulties the families experienced:

“After she passed away, just thinking about … what her mother was going through and what her stepfather was going through and what her like, final last few days were like. I think that was very, very challenging for me… Yesterday she was fine and talking and singing Mamma Mia and now she was in the PICU fighting for her life kind of thing. And seeing someone you care about and have developed a relationship within that situation is extremely challenging regardless of if it’s even a short relationship.” (MS01, post interview)

These students described how shocking and sudden such experiences were and how challenging it was emotionally, even after only knowing their buddies for a short time.

### Developing deep connections with buddies and their families is rewarding

#### Pre-match interviews

Students expressed the desire to connect with their buddies and worried about their abilities to connect initially, including how to find common interests and gain parental trust. Students echoed statements like the following: “I think finding common ground to start, making sure she’s comfortable and just making sure we have something to talk about and fun things to do might be hard. You know, the start of a relationship” (MS01). Some students explained that this was because their buddies would be much younger than other children with whom they had worked. Others with older buddies worried about being well-liked, especially if their buddies already had other friends coming to visit. Many worried “potentially the connection, maybe you like- you don’t like [each other], maybe your, um, partner doesn’t like you or like- you just, like- the fam- doesn’t match up, so like that can be stressful” (MS03). Further, students voiced concerns about finding time to spend with their buddies, which would in turn affect their abilities to connect and form meaningful relationships. They acknowledged that their medical school schedules are busy, and that their buddies’ treatment protocols may not align with their availabilities. Without enough time, students worried that they would not be able to form close, meaningful relationships and provide sufficient support. However, students did explain that they were motivated to show families that they were committed to being there as much as possible.

#### 4-month and post interviews

Students described that it was difficult to warm up to families and that they initially felt they were more of a burden than a help to them: “In the beginning the parents were pretty reserved. They were going through a rough time, you can tell like they weren’t um, as welcoming to outsiders in their care” (MS09, post interview). Expanding on that description, other students highlight how,

“At the beginning there was the whole adjustment period of them getting to know me, me getting to know them…. as things got more comfortable, then they would feel more comfortable leaving, and I would feel more comfortable watching him by [my]self.” (MS10, post interview).

Students recognized how busy the families’ schedules were and they were nervous about occupying more time, especially when the relationships were new. However, once they had time to develop rapport and spend time together, students thought the partnerships were rewarding for both themselves and their buddies’ families:

“I was very shocked to see and very happy and grateful that they actually opened up to me and we would be texting all the time. And they invited me to this close personal event. Like it’s-it just really shows that they, they welcomed me into their family.” (MS09, post interview).

Students felt lucky to participate in the program as they learned from the experiences, witnessed the resiliency of children despite intense medical therapies, and felt they were making a difference, especially when their buddies were excited to see them. Even though students initially expressed worry about finding time to visit, they were able to find the time and prioritized visits over other obligations. They found it to be a welcomed break from traditional learning experiences. Students endorsed how parents valued the time they took to spend with their children and how much their children’s moods improved when the students came for visits. Some parents explained to students that their buddies would ask for them when they were not around. As a student recounted, “I show up and he’ll cheer and be excited. And then when his poor nutritionist shows up, he gets really upset and covers his face with his pillow” (MS14, 4-month interview). Students noted that this positive impact was significant, as it allowed the children to play and forget about their diagnoses during their visits.

### Gaining Empathy and Personal Fulfillment from Buddies and their Families is Inevitable

#### Pre-match interviews

The students noted that they would aim to provide the buddies with experiences that they themselves would hope to be shown if they were in the same situation. When deciding how to interact with their buddies they anticipated, “think[ing] of any time that [they] ever felt down… then remember[ing] that role [that someone played for them] in similar situations (MS04). Students said that empathy was a personal motivator for joining the program, and they frequently expressed wanting to support others as they were often happiest when doing so. Some students expressed that this happiness comes from a sense of personal fulfillment when assisting others through stressful situations, both emotionally and practically. For instance, a student explained,

“I’ve always liked interacting with people and finding that I feel most fulfilled when I’m engaging with someone and, um, helping- helping them deal with whatever they’re going through, or just spending time with them...being a source of comfort for them, and doing whatever they need to do to help themselves feel, like, okay in that moment…. just based on previous interactions I’ve had that I’ve felt that that’s where I feel, like, really positive experiences.” (MS06).

Many students also had prior experiences working with children and families in mentorship roles and recalled those experiences to draw parallels with this program: “I have um, worked with kids before and so I know how hard sometimes it can be working with the parents during challenging times (MS15). Conversely, others reflected on their personal experiences to prepare them for the role, “I know, like, my sister was sick in the hospital when she was little…. I saw how important and what an impact…just cheering up, patients who are kids can- can be” (MS14). They described their previous experiences positively, explaining that it brought happiness to themselves and the children involved, and they predicted their partnerships in this program would generate similar emotions and experiences.

#### 4-month and post-match interviews

Students believed the program was a valuable learning experience and allowed them to gain empathy and personal fulfilment. As a student summarized, “I get an appreciation for how difficult it is for patients to be going through their illnesses” (MS14, 4-month interview). It taught them how resilient children can be despite adversity. It also taught them how to be more adaptable and flexible with scheduling visits, especially when others are balancing stressful events. Students learned to put personal conflicts into perspective, stating that their problems are not always life-or-death matters and that stressors in their lives may be miniscule in comparison to others’:

“I think it’s taught me that sometimes I’ll be complaining or upset about … some little, minor thing that’s inconvenient that’s happened to me, but then you see people who are going through one of the most difficult times in their life, and they’re not complaining and they’re staying positive…. just having that model in my life has translated to me …. thinking that there’s people going through a lot worse that are continuing and living on, so you can too.” (MS06, 4-month interview)

Students also explained how these lessons were relatable to their career and personal development. For example, they noted, “how to be … flexible in that you can’t really predict how someone is going to react in a certain situation” (MS04, 4-month interview) as well as “how important it is to kind of connect with human beings…. And to see what it’s like to really bond to someone who’s also your patient (MS07, post interview). Lastly, they voiced that working with children with serious illnesses is a reminder that treating patients is more than simply treating the disease, that medicine requires creativity and adaptability, and that one’s personal struggles may be more manageable than initially thought.

## Discussion

In this study, we sought to explore the perceptions and experiences of medical students in a pediatric buddy program. Non-medical experiences with patients can be beneficial for medical students’ provision of humanistic care. Although researchers have described other similar programs [5–8, 12, 14], the design of this study allowed us to describe students’ perceptions and experiences over time. Initially, the students established their expectations of the program experience based on their previous perceptions of and experiences from working with children or from their personal life experiences. After four months in the program, they were able to confirm their anticipated perceptions of and experiences in the program. They were also able to clearly establish meaningful connections with the program’s beneficiaries, learn to navigate families’ apprehensions, and gain an appreciation for living life to its fullest. The students’ perceptions and experiences in the program remained similar between the 4-month and post interviews, which suggests that even a short time in a partnership program can have lasting and beneficial influences on students’ perceptions and learning, especially with regards to humanistic care. We believe that our study is the first to explore the perceptions and experiences of medical students in a buddy program over time. Thus, our results advance the current literature while allowing for future studies to examine student perceptions and experiences in similar programs, with different populations, or over a longer period.

This study re-affirmed the established benefits of buddy programs for medical students [7, 8, 12, 14]. Previous studies have shown that immersive, non-medical interactions between medical students and patients with complex care needs are essential for teaching students the importance of well-rounded care, including care that addresses patients’ psychosocial needs [5–8]. The students in this study affirmed that the program was an important experience and that they learned valuable lessons, including the paramount importance of empathy, that they will apply in future patient encounters. They noted that they established meaningful connections with their buddies while providing support for the families that went beyond initial expectations, which they can bring to future patient encounters as both inspiration and motivation. The students also described learning about their own flexibility and adaptability. They learned that their personal struggles may be more manageable than initially perceived and that their experiences in the program helped them in personal and career growth. The students also learned that personal resiliency plays a role in providing well-rounded care. In a career as demanding as medicine, this lesson in resiliency may prove to be quite beneficial. These experiences further support that patients should be involved in educating clinicians to improve humanistic care [8, 12]. The consistencies between the lessons learned in this program and other similar programs, as well as their applications to the provision of humanistic care, suggest that these programs can play an important role in medical education. This study may help advocate for the creation of similar patient-student partnership programs at other centres that allow medical students to experience the psychosocial challenges of a variety of patient groups and learn important skills for providing holistic care.

The students in this study reported that while their perceptions of and experiences in the program were not without challenges, they were paramount for emotional growth and resiliency development, and may help prepare them for difficult scenarios as future physicians. The challenges described are consistent with those previously identified, as working with children with serious or life-threatening illnesses can be emotionally straining given the inherently sad nature of the loss of friendships [7, 8, 14]. This reality highlights that medical curricula should emphasize teaching communication skills, understanding the complex social needs of patients, and developing resiliency in medical students. This study may help inform the development and implementation of new learning objectives or non-didactic teaching sessions to explore these lessons.

The findings from this study suggest that the program’s initial information sessions are effective at establishing preliminary expectations among the students, as many accurately predicted their main roles and anticipated challenges based on information from these sessions, in combination with their personal values, experiences, and motives. The 4-month and post interviews revealed that students’ perceptions and experiences in the program were important for their personal and professional growth. The similarities of discussion between the 4-month and post interviews suggest that the students experienced many of the program’s benefits and educational value early, yet the perceptions and experiences became more profound as the program continued. Thus, the longitudinal design of this study highlighted the nuances of complex relationship development while also validating that the structure of the program is sufficient to form meaningful connections and teach important lessons. Further studies evaluating partnership programs should thus use a longitudinal design to effectively evaluate intricate relationships. As well, when new partnership programs are created, emphasis should be placed on allowing sufficient time for relationship formation.

One limitation to this study is a likely sampling bias for medical students with a predilection towards activities involving children as well as those with specific interests in paediatrics, as participation was voluntary and emphasized working with children associated with a pediatric academic hospital. Given the demanding time constraints and competing priorities of medical school, it is likely that there are students without a preference for working with children who did not register to be involved in the program. These students may likely still benefit from similar life experiences in similar programs, as the lessons learned in this study may not be specific to working with children and can potentially be extended to other patient interactions. Our group also included only those who identified as female. While it is possible that the findings may be influenced by gender, the partnership program has historically had a larger proportion of those who identify as females. Therefore, this ratio likely depicts an accurate picture of the overall perceptions and experiences of the participants and is reflective of the proportion of Canadian staff working in pediatrics [15]. However, since we did not collect socio-demographic information from the participants, we do not know if students’ perceptions and experiences varied by, for example, their backgrounds or ethnicities. Finally, the sample size of our final interviews was reduced to *N* = 8 (53%) students, compared to the initial *N* = 15 students. However, all participating students had completed a longitudinal match with their pediatric buddies. Due to the complexity of patients’ health and treatment protocols, not all students completed the final interview, as some matches extended well beyond the study timeframe.

## Conclusions

Tailored one-on-one partnerships between medical students and pediatric oncology patients can play an important role in medical students’ lives. This study provides a description of medical students’ expectations and opinions about such a program. Future research exploring these perceptions and experiences should use a similar longitudinal design, as it enables an understanding of changes within participants over time and the development of detailed understandings of the phenomena between researchers and participants [16]. An examination of both patient and parent perspectives, in addition to those of medical students, can further inform the meaningful impacts of these partnership programs, and may allow for comparison between programs and respective participants. When embarking on such studies, it will be important to actively involve patients and parents in the study design. Such involvement can ensure that the studies reflect the priorities and values of these participants as well as facilitate the development and use of relevant and appropriate data collection tools and processes. This information combined with the findings from the present study can inform program improvement, the development of similar programs at other institutions, and enhancements to medical school curricula.

## Data Availability

The datasets used during the current study are available from the corresponding author on reasonable request.
